# The effectiveness of three different 7 + 3 induction regimes in China: A retrospective analysis in adult patients with acute myeloid leukemia

**DOI:** 10.12669/pjms.37.1.2563

**Published:** 2021

**Authors:** Peng Lin, Boliang Zhou, Haiying Yao

**Affiliations:** 1 Peng Lin, Department of Hematology, Baoding No.1 Central Hospital, Baoding, Hebei, China; 2 Boliang Zhou, Department of General Surgery, Baoding No.1 Central Hospital, Baoding, Hebei, China; 3 Haiying Yao, Department of Hematology, Baoding No.1 Central Hospital, Baoding, Hebei, China

**Keywords:** Acute, Daunorubicin, Idarubicin, Leukemia, Myeloid, Treatment Outcome

## Abstract

**Objectives:**

In China, for economic reasons, induction regimes for acute myeloid leukemia (AML) often involve domestically produced idarubicin (IDA) rather than imported IDA. Our objective was to compare the effectiveness of induction regimens in combination with cytarabine; involving imported or domestic IDA, or daunorubicin (DNR).

**Methods:**

The study was conducted from 1^st^ July 2012 to 30^th^ November 2015 at Baoding No.1 Central Hospital. This was a retrospective cohort study of patients with newly diagnosed AML admitted to Baoding First Central Hospital, China. Patients were divided into three groups according to their treatment regimen: the IA-imported group, the IA-domestic group, and the DNR group. Clinical data, complete remission (CR), partial remission (PR), non-remission (NR) rates, and side effects were compared.

**Results:**

Total 282 patients were enrolled, including 123 patients in the IA-imported group, 98 in the IA-domestic group and 61 in the DNR group. The IA-domestic and IA-imported groups’ remission rates were similar (P=0.123) but significantly different from the DNR group (both P<0.05). Side effects were similar in all three groups and no severe side effects were reported.

**Conclusion:**

Cytarabine induction regimens showed similar remission rates in combination with IDA produced in China compared to imported IDA and were more effective than DNR.

## INTRODUCTION

Acute myeloid leukemia (AML) is a clonal malignant proliferative disease of myeloid primordial cells in the hematopoietic system.^1^AML accounts for around 80% of cases of acute leukemia in adults.^2^ The incidence of AML in the USA ranges from three to five cases per 100,000 populations.^3^ AML is a highly heterogeneous disease group.^4^ Whole genome sequencing has identified several recurrent somatic mutations that are implicated in different types of AML. More than 95% of patients with AML harbor at least one somatic mutation.^5^ AML results from abnormal proliferation of primitive and immature myeloid cells, known as abnormal blasts, in bone marrow that interfere with normal hematopoiesis.^6^ If untreated this quickly leads to bone marrow failure and death.^6^ Diagnosis is based upon the bone marrow or peripheral blood having more than 20% blasts of myeloid lineage.^7^ AML blasts can also infiltrate organs after entering the peripheral blood. Most patients have hematological abnormalities at first diagnosis. The severity of AML is usually related to the malignant proliferation of bone marrow leukemia cells. The clinical manifestations are anemia, hemorrhage, infection and fever, organ infiltration, and metabolic abnormalities.^1^ In younger patients with AML, improvements in supportive care mean 5-year overall survival (OS) has improved to around 40%-50%. However, most patients with AML are over 60 years old, and their long-term outlook is only 10%-20% 5-year OS.^8^

In clinical treatment, most patients with newly diagnosed AML receive a combination of standard-dose cytarabine with an anthracycline (daunorubicin [DNR] or idarubicin [IDA]), the so-called 7 + 3 regimen.^9^ This is then followed by high-dose cytarabine (HiDAC) consolidation for favorable-risk patients, or allogeneic stem-cell transplant (SCT) for patients with adverse-risk disease in first remission.^9^ In terms of first-line treatment, cytarabine and DNR can achieve 60-80% complete remission (CR) rates for adults, but they have short survival rates and drug resistance is a problem.^10^ Since 1990, when IDA was approved by the Food and Drug Administration (FDA) for combined chemotherapy of AML, a more satisfactory clinical therapeutic effect has been achieved, with a CR rate around 75%-85%.^11^ However, even when patients with AML achieve CR there is a high chance of relapse due to the presence of minimal residual disease.^12^ A number of targeted therapies and therapies based on the differential pathophysiology of leukemic stem cells compared with normal counterparts have emerged, but their development and testing in clinical trials to approval has taken a long time.^13^ New agents were only approved in the US in 2017.^14^ So, outside of clinical trials many patients are still treated with a regimen last updated in the 1990s.^13^

In China, due to the high price of imported IDA and the economic tolerance of Chinese patients, domestic IDA (Ainuoning, Hisun Pfizer Pharmaceutical Co., Ltd., approval No.: H20050144) is widely used in clinical practice as well as imported IDA (Zavedos, Pfizer Pharmaceutical Co., Ltd., approval No.: H20110076). But it is unclear how these different agents compare in terms of patient response. As it will take some time for most patients in China to be treated with personalized therapy for AML this information will help Chinese clinicians select optimal treatment regimens for their patients.

Therefore, the aim of the present study was to investigate the therapeutic effect and toxicity of IA (IDA; cytarabine) and DA (DNR; cytarabine) regimens in the treatment of AML in China.

## METHODS

### Ethical approval

The study was approved by the Institutional Ethics Committee of Baoding No.1 Central Hospital at March 17, 2020, and written informed consent was obtained from all participants. This was a retrospective cohort study of patients with newly diagnosed AML who were admitted to the Depart

### The inclusion criteria were:

1. ≥ 18 years old.
2. Patients who were newly diagnosed as AML according to the Diagnostic Criteria and Therapeutic efficacy of Hematological Diseases.^15^
3. Eastern Cooperative Oncology Group (ECOG) physical condition score ≤ 2 points.
4. Expected survival > 3 months.

### The exclusion criteria were:

1. Patients with acute promyelocytic leukemia (M3) according to the World Health Organization (WHO) classification^16^ after bone marrow morphology, immunophenotype, cytogenetic, and molecular biology (MICM) classification.
2. Patients with other malignant tumors or had undergone radiotherapy or chemotherapy within 5 years.
3. patients with severe organ dysfunction or disease (such as severe cardiopulmonary disease or severe hepatorenal insufficiency).
4. Pregnant women.

All of the patients were divided into three treatment groups according to the treatment they received: those treated with IDA imported by Zavedos combined with cytarabine chemotherapy were the IA-imported group, those treated with domestic IDA manufactured by Ainuoning combined cytarabine with chemotherapy were the IA-domestic group, and those treated with DNR and cytarabine combined chemotherapy were the DNR group.

### Treatment

All patients completed at least one course of differentiation induction therapy. The IA regimen was as follows: IDA: 8-10mg/m^2^ daily for 3d, administered by intravenous drip; cytarabine: 100-200mgm^2^ daily for 7d, administered by intravenous drip twice for 4-6 hours. The DA regimen was as follows: DNR: 45-60mg/m^2^ daily for 3d, administered by intravenous drip; cytarabine: 100-200mg/m^2^ daily for 7d, administered by intravenous drip twice for 4-6 hours. If leukocyte count in the peripheral blood of patients before chemotherapy was higher than 50×10^9^/L, low dose of cytarabine or hydroxyurea was given orally to reduce leukocytes and prevent tumor lysis syndrome. All three groups were treated with one course of IA or DA regimen induction therapy to judge the therapeutic effect.

During chemotherapy, fluid infusion and nutritional support were given, meanwhile, gastric mucosa protection, antiemetic, liver protection, hydration and alkalization were applied. Patients with elevated serum uric acid were treated with sodium bicarbonate or allopurinol to reduce uric acid. Changes in blood routine were monitored. When hemoglobin (HB) was < 60g/L, suspended red blood cell transfusion was given to correct anemia; when platelet (PLT) count was < 10×10^9^/L or hemorrhagic tendency occurred, platelet transfusion alone was given to enhance platelet count; when granulocyte deficiency (NEUT<0.5×10^9^/L) occurred 48 hours after discontinuation of chemotherapy, subcutaneous injection of granulocyte colony-stimulating factor (G-CSF) was given; anti-infection treatment was given to patients with infection according to the reports of imaging and bacteriology.

### Examinations

At the time of initial diagnosis, routine blood analysis, liver and kidney function, myocardial enzymes, coagulation function, bone marrow puncture, biopsy, chest computed tomography (CT), electrocardiogram, abdominal ultrasound or CT examination were determined, and bacteriological examinations such as throat swab, nasal swab, perianal swab were carried out to determine whether there were colonizing bacteria present. During the chemotherapy course, routine blood tests were monitored daily or once every two days, liver and kidney function was monitored weekly, and coagulation function was monitored when necessary. Bone marrow puncture was performed on the day of cessation of chemotherapy, 7-10 days after the cessation of chemotherapy, and after the recovery of hemogram.

### Clinical data collection and follow up

According to the criteria for diagnosis and treatment of hematological diseases^15^, the clinical therapeutic effect evaluation was divided into CR, partial remission (PR) and non-remission (NR) according to peripheral hemogram, bone marrow images and corresponding laboratory examinations. Safety evaluation was carried out according to WHO adverse reaction evaluation criteria for anticancer drugs. Duration of granulocyte deficiency refers to the time from granulocyte deficiency occurred to neutrophil > 0.5×10^9^/L, and platelet recovery time refers to the time from PLT<50×10^9^/L after chemotherapy to PLT>50×10^9^/L. All the patients were followed up until 20^th^ December 2015. Based on the criteria of the NCCN guidelines^17^, patients were divided into good, moderate and poor prognosis groups according to their cytogenetic data (Table-I).

**Table-I:** Prognostic grouping of AML (non-APL)

Prognostic grouping	Cytogenetics
Good	inv (16) ,t (8; 21) , t (16; 16)
Moderate	Normal karyotype, +8, t (9;11),other unfavorable and adverse abnormalities
Poor	Complex karyotype (≥3 abnormalities) -5, -7,5q-, 7q-, 11q23 abnormalities except t (9; 11), t (3; 3), t (6; 9), t (9; 22)

### Statistical analysis

SPSS19.0 software (IBM Corp., USA) was used for the statistical analysis of clinical therapeutic effect. Enumeration data were expressed by quantity (percentage), and Chi-squared test was used for the comparison among the three groups. Measurement data conforming to normal distribution were expressed by mean± (standard deviation) SD, and analysis of variance (ANOVA) with post hoc test was used for comparison among the three groups; measurement data that did not conform to normal distribution were expressed by median (range), and Kruskal-Wallis test was used for comparison among the three groups. *P*<0.05 was considered to be a significant difference.

## RESULTS

A total of 282 newly diagnosed AML patients were enrolled, including 123 patients in the IA-imported group, 98 in the domestic group and 61 in the DNR group. The proportion of male patients was 60.98% in the IA-imported group, 42.86% in the IA –domestic group and 47.54% in the DNR group (P=0.021). There was no significant difference in age, leukemia type, peripheral hemogram, or prognostic grouping among the three groups (all P>0.05) (Table-II).

**Table-II:** Comparison of clinical features before initial treatment among the three groups.

	IA-imported group	IA-domestic group	DNR group	P value
No. of cases	123	98	61	
***Gender, n (%)***				0.021
Male	75 (60.98%)	42 (42.86%)	29 (47.54%)	
Female	48 (30.02%)	56 (57.14%)	32 (52.46%)	
Median age, (years)(median, range)	42 (14~76)	43 (14-76)	43 (15~74)	0.981
***AML subtype***				0.468
M0	0 (0%)	1 (1.02%)	0 (0%)	
M1	7 (5.69%)	2 (2.04%)	2 (3.28%)	
M2	77 (62.60%)	55 (56.12%)	37 (60.66%)	
M4	19 (15.45%)	24 (24.49%)	16 (26.23%)	
M5	18 (14.63%)	14 (14.29%)	5 (8.20%)	
M6	2 (1.63%)	1 (1.02%)	0 (0%)	
M7	0 (0%)	1 (1.02%)	0 (0%)	
***Hemogram, median (range)***				
WBC (×109/L)	40.6 (0.7-442.1)	40.7 (0.3-359.1)	35.9 (0.8-232.0)	0.687
HB (g/L)	77.3 (40-157)	81.0 (42-136)	77.0 (35-220)	0.725
PLT (×109/L)	61.8 (3-346)	62.6 (3-383)	64.1 (2-559)	0.869
***Prognostic grouping***				0.075
Good	34 (27.64%)	23 (23.47%)	15 (24.59%)	
Moderate	71 (57.72%)	71 (72.45%)	37 (60.66%)	
Poor	18 (14.63%)	4 (4.08%)	8 (13.11%)	

In the IA-imported group, the CR rate was 85.4% after a single course of treatment, PR rate was 10.6%, and NR rate was 4.1%; in the IA-domestic group, the CR rate was 75.5% after a single course of treatment, PR rate was 20.4%, and NR rate was 4.1%; in the DNR group, the CR rate was 57.4% after a single course of treatment, PR rate was 37.7%, and NR rate was 4.9% (Table-II). These rates were significantly different between the three groups (P<0.05), but no significant difference was found between the IA-domestic and IA-imported groups (P=0.123) when the groups were compared in a pairwise manner. Both IA groups were significantly different from the DNR group by pairwise comparison (P<0.05) as seen in Table-III.

**Table-III:** Comparison of remission rates among the three groups.

	IA-imported group	IA-domestic group	DNR group	P value
No.	123	98	61	
CR, n (%)	105 (85.4%)	74 (75.5%)	35 (57.4%)	
PR, n (%)	13 (10.6%)	20 (20.4%)	23(37.7%)	<0.05
NR, n (%)	5 (4.1%)	4 (4.1%)	3(4.9%)	

P=0.123: IA-imported group vs. IA-domestic group, P<0.05: IA-imported group vs. DNR group, P=0.049: IA-domestic group vs. DNR group.

IDA is commonly used at a dose of 8-12 mg/m^2^. In this study, a dose of 8-10 mg/m^2^ was adopted. The CR rate was 77.6% in 112 patients treated with a dose of 8 mg/m^2^, and 84.4% in 109 patients treated with a dose of 10 mg/m^2^. In the IA-imported group, 53 patients were treated with a dose of 8 mg/m^2^, and the CR rate was 84.9%, 70 patients were treated with a dose of 10 mg/m^2^, and the CR rate was 85.7% (P=0.378). However, in the IA-domestic group, the CR rate of patients treated with a dose of 10 mg/m^2^ was significantly higher than that of patients treated with a dose of 8 mg/m^2^ (82.1% vs. 71.2%, P=0.021). The CR rate of patients treated with either a dose of 8 mg/m^2^ or 10 mg/m^2^ was similar in the two IA groups (all P>0.05) (Table-IV).

**Table-IV:** Remission rates at different doses between IA-imported and IA-domestic groups.

Dose	IA-imported group	IA-domestic group	P value
8mg/m2			
No.	53	59	
CR (%) 10mg/m2	45 (84.9%)	42 (71.2%)	0.082
No.	70	39	
CR (%)	60 (85.7%)	32 (82.1%)	0.614

IA-imported group: 8 mg/m2 vs. 10mg/m2, P=0.378 IA-domestic group: 8 mg/m2 vs. 10mg/m2, P=0.021.

### Side-effect evaluation

Different degrees of bone marrow suppression occurred in patients of all three groups after chemotherapy. The proportions of patients with neutrophil deficiency and thrombocytopenia after chemotherapy were 100% in all the three groups. No significant differences were found in minimum median granulocyte count (P=0.058), the duration of granulocyte deficiency (P=0.878), the minimum median platet count (P=0.351) and the recovery time of thrombocytopenia (P=0.317) among the IA-imported, IA-domestic and DNR groups (Table-V).

**Table-V:** Side effects in the three patient groups during treatment and follow-up.

	IA-imported group	IA-domestic group	DNR group	P-value
Granulocyte deficiency, n (%)	123 (100%)	98 (100%)	61 (100%)	
Minimum granulocyte count, median (range)	0.02 (0-0.39)	0.02 (0-0.23)	0.04 (0-0.6)	0.058
Duration of granulocyte deficiency, median(range)	10 (4-36)	11 (6-27)	9 (1-31)	0.878
Thrombocytopenia, n (%)	123 (100%)	98 (100%)	61 (100%)	
Minimum platelet count, median (range)	5 (2-21)	6 (1-19)	9 (1-23)	0.351
Recovery time of thrombocytopenia, median(range)	10 (0-42)	13 (7-30)	13 (6-27)	0.317
***Infection sites, n (%)***				
Lungs	39 (31.7%)	34 (34.7%)	19 (31.1%)	0.912
Upper respiratory tract	27 (21.9%)	22 (22.5%)	11 (18.0%)	0.836
Intestinal tract	11 (8.9%)	7 (7.1%)	4 (6.6%)	0.817
Perianal	16 (13.0%)	13 (13.3%)	11 (18.0%)	0.907
Skin and soft tissue	9 (7.3%)	6 (6.1%)	4 (6.6%)	0.985
Blood	12 (9.8%)	13 (13.3%)	9 (14.8%)	0.733
Urinary system	4 (3.3%)	1 (1.0%)	2 (3.3%)	0.971
Others	5 (4.1%)	2 (2.0%)	1 (1.6%)	0.651

In terms of infection, different degrees of infection symptoms appeared in patients of the three groups. The sites of infection were pulmonary, upper respiratory tract, intestinal tract, perianal, blood flow infection, skin, and soft tissue infection. They were not significantly different among the three groups (all P>0.05). The infection symptoms were controlled after timely anti-infection treatment.

In terms of non-hematological side effects after treatment, all patients had adverse reactions such as nausea and vomiting to varying degrees, but they were able to tolerate chemotherapy after the use of antiemetic drugs; drug-induced dermatitis occurred in one patient in the IA-domestic group, and it was improved after symptomatic treatment; no severe side effects such as impairment of liver and kidney function, arrhythmia and heart failure occurred in patients of any of the three groups.

## DISCUSSION

This study compared the effectiveness of induction regimens in combination with cytarabine; involving imported or domestic IDA, or DNR. Because many Chinese patients with AML choose domestic IDA for economic reasons, information on the effectiveness of this drug compared to standard therapies is important to ensure that patients are receiving the best treatment possible. The results show that in terms of remission rates the two different forms of IDA provided similar outcomes and they were both more effective than DNR. The safety of all three induction regimens was similar.

The results of this study support the established view that IDA produces favorable remission results compared to DNR. The CR rate of imported IDA was 85.4% and the CR of domestic IDA was 75.5% compared to a CR of 57.4% with DNR. These results compare well with other studies.^18^-^20^ The CR rate of DA regimen with standard dose is around 60%-80% in the initial treatment of adult AML patients, while that of the IA regimen can reach 70%-85%^18^ A Cochrane review in 2015 concluded from the results of 27 randomized controlled trials that IDA in induction therapy of newly diagnosed AML, prolongs OS and DFS, increases CR rate and reduces relapse rate compared to DNR, although it also increases the risks of death on induction therapy and grade 3/4 mucositis.^21^ Therefore, use of IDA should be carefully considered in patients less able to tolerate treatment. However, a recent study in elderly patients found that reduced intensity IDA plus cytarabine therapy resulted in 70.4% CR compared to 40% CR for those treated with DNR plus cytarabine.^22^ IDA was developed by removing one OCH group from the fourth carbon element in the anthracycline structure of DNR. This change increases the liposolubility of IDA, enhances its biological activity, and means it can penetrate the cell membrane faster and more easily, increasing absorption by leukemia cells, and prolonging its half-life. IDA intercalates double-stranded DNA in vivo, blocks nucleic acid synthesis, and inhibits DNA elongation, leading to cell death; its concentration in cells is significantly higher than that in plasma, and the metabolite of IDA (IDAol) also has a strong cytotoxic effect, which greatly prolongs its clearance in vivo. Because IDA does not induce the expression of P-glycoprotein (p-gP), drug resistance is rarely acquired.^20^,^23^ However, in this study while we found a higher remission rate with IDA there was no expected increase in side effects.

In the IA-imported group, the minimum median granulocyte deficiency was 0.02×10^9^/L, and the median duration was 10 days; in the IA-domestic group, the minimum median granulocyte deficiency was 0.02×10^9^/L, and the median duration was 11 days; moreover, in the DNR group, the minimum median granulocyte deficiency was 0.04×10^9^/L, and the median duration was nine days. Granulocyte deficiency was found in all the three groups after chemotherapy, and it lasted for more than a week, so there was a greater chance of infection. Studies have reported that compared with DNR, the induction therapy of AML with IDA had obvious bone marrow suppression, and it lasted for a long time.^24^ In this study, there was no significant difference in the degree or duration of bone marrow suppression among the three groups. These differences may be due to the relatively small sample size in this study. The analysis of infection situation showed similar infections in all three groups, so the roles of timely and effective anti-infective treatment and comprehensive supportive treatment should be paid attention in any chemotherapy program.

Due to the high price of imported IDA, the clinical application of IDA is limited to a certain extent. In this study, domestically produced IDA also achieved a good therapeutic effect, and the toxicity and side effects of the three induction regimens were similar and tolerable, so they can be widely used in clinical practice. IDA is commonly used at a dose of 8-12 mg/m^2^. In this study, a dose of 8-10 mg/m^2^ was adopted. In the IA-imported group, dose changes had no significant effects on CR rate, but in the IA-domestic group, the CR rate of patients treated with a dose of 10 mg/m^2^ was significantly higher than that of patients treated with a dose of 8 mg/m^2^. This result shows that there are some differences in treatment with the two forms of IDA and that the higher dose should be considered when clinicians are using domestic IDA. In terms of hematological toxicity, there was no significant difference in patients treated with different doses between the IA-imported and IA-domestic groups.

### Limitations of the study

The number of patients in each of the three groups was quite small. Because of short follow-up time, the OS and DFS time of the three groups were not compared, so long follow-up time and large sample size are needed in further studies.

## CONCLUSION

In this study, domestically produced IDA achieved a comparable therapeutic effect to imported IDA and both were more effective than induction that involved DNR for treatment of patients with newly diagnosed AML. The toxicity and side effects of the three induction regimens were similar and tolerable.

### Authors’ Contributions

**PL and HY** carried out the studies, participated in collecting data, drafted the manuscript, and are responsible and accountable for the accuracy or integrity of the work.

**BZ** performed the statistical analysis and participated in its design.

**PL and** **BZ** participated in acquisition, analysis, or interpretation of data and draft the manuscript.

All authors read and approved the final manuscript.
